# The efficiency of otoacoustic emissions and pure-tone audiometry in the detection of temporary auditory changes after exposure to high sound pressure levels

**DOI:** 10.1016/S1808-8694(15)30118-X

**Published:** 2015-10-19

**Authors:** Samanta Marissane da Silva Barros, Silvana Frota, Ciríaco Cristovão Tavares Atherino, Francisco Osterne

**Affiliations:** a M.S. in Speech and Hearing Therapy - Veiga de Almeida University; b PhD in Human Communications Disorders - UNIFESP; Professor at UFRJ and UVA; c PhD in Otorhinolaryngology - USP; Professor at UERJ and UVA; d M.S. in Human Communications Disorders - UNIFESP; Professor at UVA. Veiga de Almeida University

**Keywords:** audiometry, otoacoustic emissions, temporary hearing loss

## Abstract

Exposure to noise has a harmful effect on the auditory health of workers. **Aim:** The main goal of this paper was to establish the role of pure-tone audiometry and evoked transient otoacoustic emissions in the detection of small temporary auditory changes after exposure to high sound pressure levels. Study Design: a cross-sectional cohort study. **Material and Methods:** 30 otologically normal subjects aged between 20 and 35 years were submitted to pure-tone audiometry and evoked transiente otoacoustic emissions before and after 5 hours of exposure to high sound pressure levels (betweeen 80 and 90 dB). **Results:** For pure-tone audiometry the largest changes occurred at high frequencies - from 3 KHz to 8 KHz after exposure. The evoked transient otoacoustic emissions showed reduced reproductibility from 1 KHz to 4 KHz after exposure to noise. **Conclusion:** We noted that both puretone audiometry and evoked transient otoacoustic emissions had a role in detecting statistically significant changes in the auditory threshold and in reproductibility, after exposure to high sound pressure levels.

## INTRODUCTION

Among the various occupational risk factors, noise exposure may be considered as one of the most harmful agents for the auditory health of workers.

Constant exposure to high sound pressure levels alters the physical and mental well-being of workers; the human auditory apparatus is extremely vulnerable to this occupational risk factor.^1^

Noise exposure may result in variable levels of auditory alterations as well as non-auditory problems, which affect the social, family and occupational behavior of workers.

The inner ear, specifically the sensory cells of the cochlea, is very sensitive to exposure to high sound pressure levels; the outer hair cells are the first structures to be affected.

Outer hair cells are the cochlear amplifier, amplifying sound stimuli and affecting the function of the inner hair cells, which are the true cochlear receptors and coding cells.^2^

Intense exposure to noise may cause mechanical rupture of the basilar membrane and the auditory sensory cells, resulting in immediate hearing loss of varying degrees, which in turn may produce temporary or permanent auditory threshold shifts.^3^

Hearing loss due to noise exposure is initially reversible, seen as a temporary auditory threshold shift in the 2000 to 6000 Hz range. The presence of varying degrees of temporary threshold shifts suggests susceptibility to permanent sensorineural hearing loss. This condition has been amply investigated by the scientific community.^4^

A temporary threshold shift may be defined as an increased auditory threshold that recovers gradually after noise exposure.^5^

Some authors^6^ have described temporary thresholds shifts as a short term effect, a change in auditory sensitivity that gradually returns to normal once noise exposure has ceased. Threshold variations are directly related with individual susceptibility, time of noise exposure, and sound intensity and frequency. Temporary threshold shifts are seen mostly in the 2000 to 6000 Hz range, especially if threshold shifts are associated with exposure to noise over 80 dB.

Intense noise exposure may result in altered sound perception for high frequencies.^7^ This type of dysfunction is associated with difficulty in selectively detecting a specific sound frequency in a noisy environment.

A study that attempted to characterize the noise-exposed auditory system8 in 203 subjects found that 98% of audiometries done after noise exposure showed altered configurations. The authors concluded that there is a specific stage before temporary auditory threshold shifts, as the noise-exposed auditory system presented subliminal auditory changes with no apparent injury.

Auditory monitoring of workers, usually in industry, is done using pure tone audiometry. Epidemiological surveillance authorities use this test to analyze auditory changes resulting from noise exposure.^9^

Pure tone audiometry is a subjective test frequently used to assess the acoustic sensitivity of subjects exposed to external agents that may cause hearing loss. The test, however, is affected by a number of functional and psychosocial factors; depending on the worker’s physical and emotional state, it may generate unreal and contradictory auditory responses and results.

Pure tone audiometry includes factors that cannot be controlled and that may interfere with its execution, interpretation and results. Audiometry is a subjective test where results may be altered by inherent individual factors.^10^

Occupational auditory assessments done only with pure tone audiometry may not reveal the true condition of the cochlea of a worker; this reality can undermine the prevention work done in a manufacturing facility.

Application of objective tests, such as those using evoked otoacoustic emissions (EOAEs), associated with an occupational auditory assessment, would be of significant value to monitor the hearing of workers and to analyze their susceptibility to hearing loss when exposed to elevated levels of sound pressure.

Assessment of EOAEs allows direct investigation of the outer hair cell cochlear amplification mechanism; it is an objective, non-invasive and sensitive test that provides preventive and more effective auditory monitoring.^11^

In 1978 Kemp11 noted that the cochlea was able to produce and to receive sound. The cochlear ability to generate sound received the name otoacoustic emissions (OAE). Kemp observed that this energy could be measured with little invasiveness in the external auditory canal, where the presence of OAE would suggest normal cochlear function.

OAE were divided into spontaneous and evoked otoacoustic emissions. In 1989^11^ Kemp defined spontaneous OAE as stationary signals that may be recorded without acoustic stimulation and that may be found in 50% of normal hearing subjects. EOAE would be cochlear outer hair cell responses generated by various sound stimuli. OAE have been classified as transient, distortion product and stimulus-frequency.

OAE are associated with normal cochlear and middle ear function. The presence of OAE indicates that the middle ear and the cochlea respond normally to sound stimulation. It is then possible to assess the sensitivity of the efferent auditory system after exposure to elevated sound pressure levels or to ototoxic drugs. When middle ear structural conditions are discarded, absence of OAE may be directly associated with a variably altered cochlea.^12^

Studies of EOAE^1^,^3^ done in 25 male and female subjects exposed to 100 dB NA during 10 minutes have revealed that EOAEs may detect temporary auditory threshold shifts resulting from noise exposure.

Routine OAE testing in occupational audiological assessments can increase knowledge about the inner ear pathophysiology.^14^ EOAEs may be extremely useful in evaluating individual susceptibility to noise, since emission changes may be detected before auditory tone threshold shifts occur.

Hotz et al.^15^ investigated the effect of noise in 117 recruits and 30 cadets to establish the usefulness of transient evoked otoacoustic emissions (TEOAEs) for auditory monitoring. The soldiers underwent pure tone audiometry and TEOAEs testing before and after a 17-week training period during which the subjects were exposed to elevated sound pressure levels from firearms. A comparative analysis between pure tone audiometry and transient otoacoustic emissions (TOAEs) concluded that TOAEs are more sensitive in detecting initial cochlear alterations that result from noise exposure. The authors demonstrated that testing of EOAEs is an effective clinical tool for monitoring the cochlea; advantages include ease, speed, sensitivity and specificity, allowing subtle cochlear alterations to be detected earlier compared to pure tone audiometry.

The use of EOAEs in occupational auditory assessment would be valuable to monitor the hearing of large groups exposed to elevated sound pressure levels.

The aim of this study was to investigate pure tone audiometry and TOAEs, before and after exposure to elevated sound pressure levels, to establish the efficiency of both methods in detecting minor temporary auditory threshold shifts.

## MATERIAL AND METHODS

This study was reviewed and approved by the Research Ethics Committee of the Veiga de Almeida University, under protocol number 28/04.

Thirty otologically normal male subjects aged between 20 and 35 years, with healthy external and middle ears, employed in a textile factory for at least one year and not more than three years, with no history of work done under conditions of auditory risk in any other company, were selected.

All of the workers used individual protection equipment (earmuffs or earplugs) and worked only in activities that involved exposure to elevated sound pressure levels of around 80 to 90 dB NPS.

The first stage was selection of the sample, which involved otological clinical history-taking, pure tone audiometry and tympanometry. Recording of auditory thresholds was done in an acoustic booth with appropriate sound pressure levels and ambient noise ratios for each frequency that was investigated, according to ANSI S.31 (1991) standards.

Pure tone audiometry was done using an Ad 27 Audiotest Interacustico audiometer calibrated according to ANSI S.31 (1991) standards and a TDH 39 P earphone. Thresholds were assessed at 250, 500, 1000, 2000, 3000, 4000, 6000 and 8000 Hz using a continuous pure tone stimulus ascending and descending technique.

Workers underwent tympanometry to exclude middle ear conditions. A middle ear Amplaid 775 analyzer calibrated according to ANSI S.31 (1991) standards was used.

Workers that had audiometric thresholds over 25 dB NA (ISO-1989) at one or more air conduction test frequencies in the pre-exposure to noise investigation were excluded, as were subjects with As, Ad, B and C tympanometric curves.

Audiometric tests that showed normal auditory thresholds and type A tympanometric curves became the pre-exposure to elevated sound pressure group. These subjects were sent to the second stage of the investigation.

The second stage included pure tone audiometry and recording of TOAEs before and after 5 hours of exposure to elevated sound pressure levels (80 to90 dB). The investigation had the following sequence:

before exposure to elevated sound pressure levels

- pure tone audiometry before the worker was exposed to elevated sound pressure levels; auditory rest for 14 hours (done during the sample selection stage).
- measurement of TOAEs before the worker was exposed to elevated sound pressure levels.

after exposure to elevated sound pressure levels

- pure tone audiometry after 5 hours of exposure to elevated sound pressure levels (80 to 90 dB).
- measurement of TOAEs after 5 hours of exposure to elevated sound pressure levels (80 to 90 dB).

Monitoring of cochlear function included recording of TEOAEs using a Capella/Madsen sound emitter coupled to a Compaq/Presario computer.

Recording of TOAEs was done in the acoustic booth. Analysis of TOAEs was done at the 1, 2, 3 and 4 KHz frequency range using a non-linear stimulus, a 70 dB NA click and wave stability equal to or higher than 70%.

TOAEs testing included an investigation of reproducibility before and after 5 hours of exposure to elevated sound pressure levels. The criterion for assessing the presence of a response was reproducibility equal to or higher than 70% in three frequencies (GATANU 1998).

Data analysis included calculation of central tendency measures (mean, median and standard deviation). Auditory thresholds and reproducibility of TOAEs before and after exposure to elevated sound pressure levels for the right and left ears were compared using Student’s t-test (critical t equal to or higher than 1.7) for the difference between two groups before and after exposure to elevated sound pressure levels.

## RESULTS

### Investigation of Pure Tone Audiometry

Analysis of the central tendency (mean) of auditory thresholds revealed shifts (worsened results) at all frequencies after exposure to elevated sound pressure levels for both ears. The most significant shifts were found at 8000 Hz in the right ear and at 6000 Hz in the left ear.

**Chart 1:**
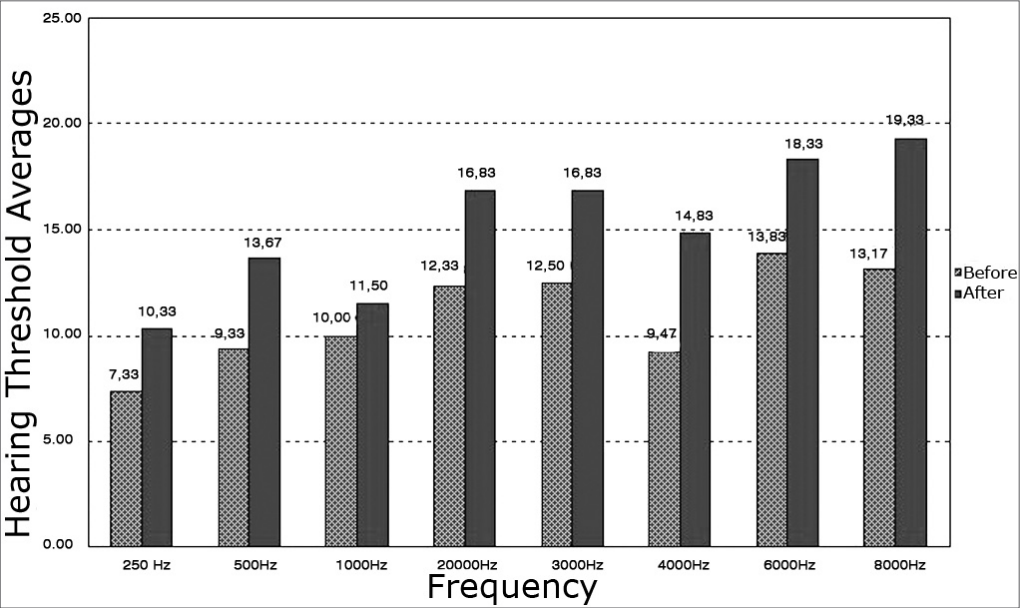
Before and after exposure to high sound pressure levels hearing threshold averages in the right ear of male subjects.

**Chart 2:**
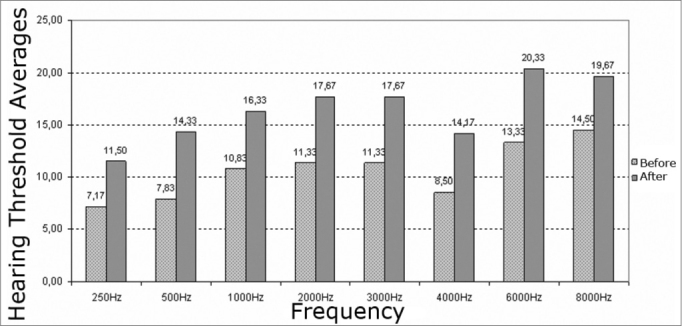
Hearing thresholds mean values and measures of central trends, average, standard deviation and median values before and after noise exposure in the left ears of male subjects.

The most significant auditory threshold shifts (for worse) were found at 4000 Hz (76% of subjects) in the right ear and at 3000 Hz (90% of subjects) in the left ear. Auditory threshold shifts varied from 5 to 20 dB in the right ear and 5 to 15 dB in the left ear (Charts 1 and 2).

**Chart 3:**
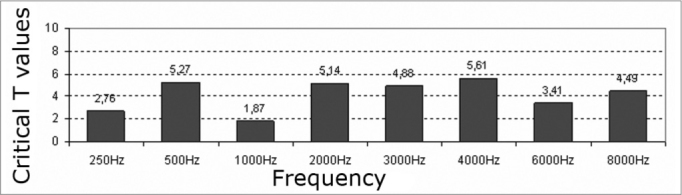
Critical T values – 1.7, significance levels of the right ear hearing thresholds.

**Chart 4:**
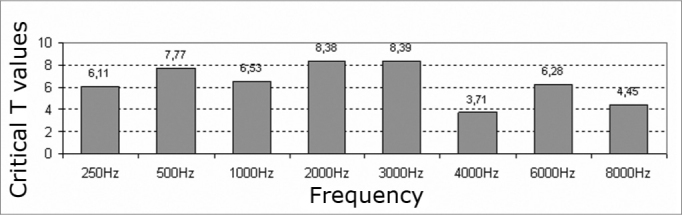
Critical T values – 1.7, significance levels of the left ear hearing thresholds.

The comparative study of auditory thresholds before and after exposure to elevated sound pressure levels using Student’s parametric t-test revealed statistically significant differences (critical t higher than 1.7) at all frequencies in both ears (Chart 3 and 4).

### Investigation of Transient Evoked Otoacoustic Emissions

**Chart 5:**
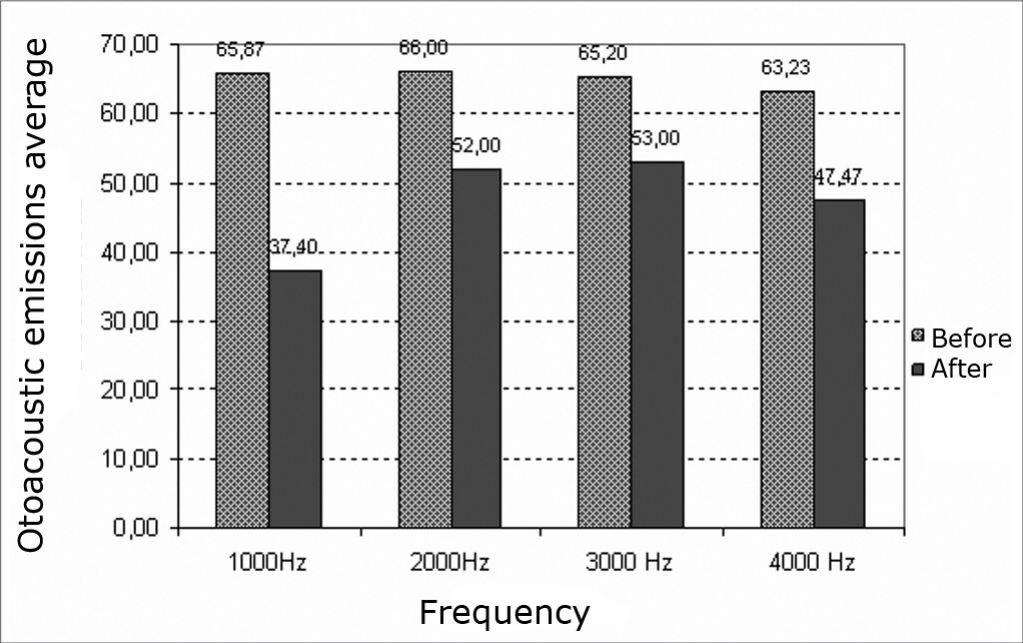
Reproducibility average of transient otoacoustic emissions before and after exposure to high sound pressures in the right ears of males.

**Chart 6:**
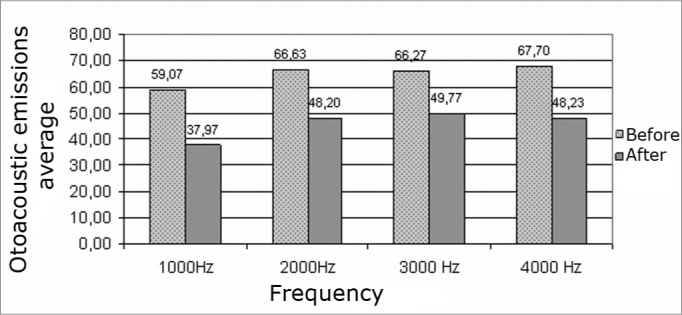
Reproducibility average of transient otoacoustic emissions before and after exposure to high sound pressures in the left ears of males.

Analysis of TEOAEs results revealed reduced reproducibility at all investigated frequencies in the right and left ears after exposure to elevated sound pressure levels (Charts 5 and 6).

Analysis of the central tendency (mean) of the reproducibility of TOAEs before and after exposure to elevated sound pressure levels revealed frequency changes (for worse) at 1000, 2000, 3000 and 4000 Hz in the right and left ears. The most significant reproducibility changes, compared to mean values, were seen at 1000 Hz in the right and left ears.

**Chart 7:**
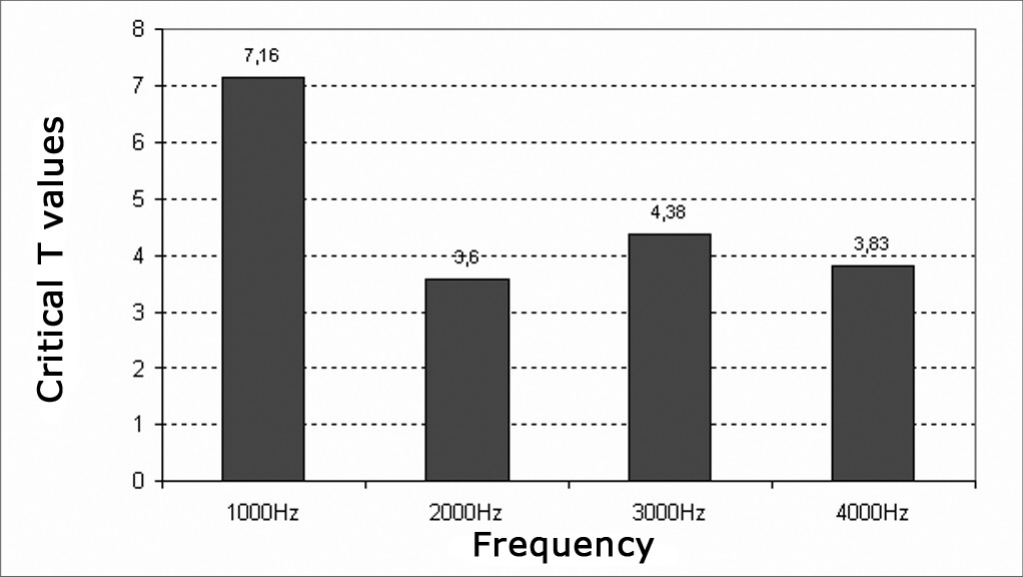
T-critical values – 1.7, Significance level of reproducibility average of transient evoked otoacoustic emissions before and after exposure to high sound in the left and right ears of males.

**Chart 8:**
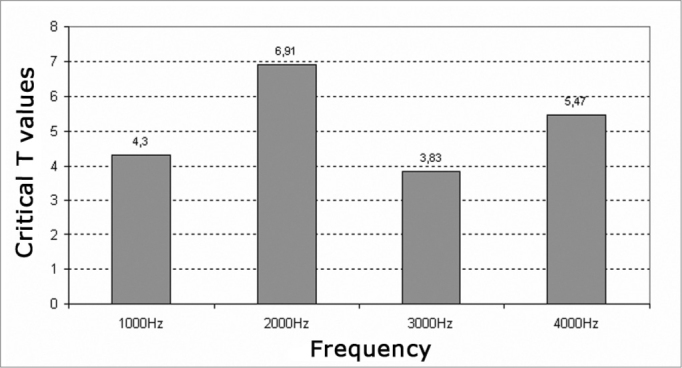
T-critical values – 1.7, Significance level of reproducibility average of transient evoked otoacoustic emissions before and after exposure to high sound in the left and right ears of males.

We found a significant decrease of reproducibility at all frequencies investigated (critical t higher than 1.7) (Charts 7 and 8).

## DISCUSSION

The analysis of individual auditory thresholds before and after 5 hours of exposure to elevated sound pressure levels revealed audiometric configuration alterations and auditory threshold shift values that agree with those found in the literature.^4^, ^6^, ^7^, ^8^, ^16^, ^17^ There was also a high incidence of significant changes at high frequencies. Studies on musicians exposed to noise^16^, ^17^ have shown auditory threshold shifts after exposure to elevated sound pressure levels; all of the participants had auditory threshold shifts, particularly at 3000, 4000 and 6000 Hz.

Another study8 that included 203 subjects exposed to noise revealed altered audiometric configurations in 98% of cases. These findings suggest that there is a specific stage before temporary auditory threshold shifts, as the noise exposed auditory system may present subliminal auditory alterations with no apparent injury.

The findings of our analysis of individual auditory thresholds, that showed the most significant auditory threshold shifts at higher frequencies, are similar to those in the abovementioned studies; the most significant changes were seen at 4000 Hz in the right ear and at 3000 Hz in the left ear.

Our findings corroborate the conclusion reached by other authors^4^, ^6^, ^7^, ^8^, ^16^, ^17^ that there is a correlation between exposure to elevated sound pressure levels and temporary auditory threshold shifts, particularly at high frequencies (3000 to 8000 Hz).

Various studies^11^, ^12^, ^13^, ^14^, ^15^ have described the importance of using TOAEs to detect subtle cochlear alterations and to monitor the hearing of individuals exposure to elevated sound pressure levels.

A comparison between our paper and similar investigations by other authors,^13^, ^14^ which studied TOAEs as a clinical tool for assessing temporary auditory threshold shifts and cochlear alterations, shows similar results for decreased cochlear reproducibility at middle and high frequencies.

A study of 25 subjects using recording of EOAEs and pure tone audiometry before and after exposure to 100 dB NA during 10 minutes revealed that an assessment of OAEs can detect temporary auditory threshold shifts. The analysis of TOAEs showed that there was a higher occurrence of worsened post-stimulus mean amplitude responses at high frequencies. This study also showed that TOAEs are more sensitive to noise exposure.

Studies that applied TOAEs on military personnel^15^ revealed that TOAEs can be a clinical tool for detecting subtle cochlear changes before they appear in pure tone audiometry. The authors found amplitude changes in TOAEs at 2000 and 4000 Hz and no change at 250 and 500 HZ.

Researchers^15^, ^13^ have stated that TOAEs allow early detection of subtle cochlear changes before they are seen in pure tone audiometry, suggesting that TOAEs are more sensitive to noise exposure, and that TOAEs may detect temporary auditory threshold shifts following exposure to elevated sound pressure levels.

These findings show that TOAEs were able to detect temporary auditory threshold shifts, as was found in our study. Our results revealed changes (reduced reproducibility) at 1000 to 4000 Hz demonstrating that TOAEs were sensitive enough to detect temporary auditory threshold shifts and cochlear alterations produced by exposure to elevated sound pressure levels, and that this exposure resulted in worsened reproducibility at middle and high frequencies.

## CONCLUSION

We concluded that both the pure tone audiometry and the transient evoked otoacoustic emissions (TEOAEs) testing were efficient in detecting minor temporary statistically significant changes in auditory thresholds following exposure to increased sound pressure levels in the right and left ears.
